# Nutrition Knowledge and Satisfaction Levels of Postbariatric Surgery Adults in the United Arab Emirates: A Pilot Study

**DOI:** 10.1155/2019/9148459

**Published:** 2019-04-01

**Authors:** Souheir Alia, Habiba I. Ali, Taoufik Zoubeidi, Mariam Ahmed

**Affiliations:** ^1^Department of Food Science, United Arab Emirates University, P.O. Box 15551, Al Ain, UAE; ^2^Nutrition and Health Department, United Arab Emirates University, P.O. Box 15551, Al Ain, UAE; ^3^Department of Statistics, United Arab Emirates University, P.O. Box 15551, Al Ain, UAE; ^4^Intern House Officer, Dubai Health Authority, Dubai, UAE

## Abstract

Research assessing the nutrition knowledge of postbariatric surgery patients is limited, although this category of patients is predisposed to malnutrition. In this pilot study, we explored postbariatric nutrition knowledge, satisfaction levels with dietitian nutrition counseling, and decision to undergo bariatric surgery of 83 patients who attended a postbariatric outpatient nutrition clinic in Dubai, United Arab Emirates (UAE). A cross-sectional design involving gender-stratified random sampling method was used to recruit 83 postbariatric surgery participants. A self-administered questionnaire was employed to collect information about nutrition knowledge related to dietary recommendations after bariatric surgery as well as participant views on dietitian nutrition counseling, their decision to undergo bariatric surgery, and nutrition-related complications experienced after the surgery. The mean (SD) knowledge score of postbariatric diet was 9.7 (2.05) out of a maximum possible score of 14. The majority of the participants (78.3%) correctly identified which foods are recommended during the first stage of the postbariatric surgery diet, and more than 90% knew about the importance of high-protein supplements after bariatric surgery. Female participants had significantly higher mean knowledge score compared to males (*p*=0.02). Although nearly 80% of the participants reported regular follow-up with their dietitian, only 10.8% reported high adherence to the dietitian's instructions. Moreover, more than two-thirds of the participants (71.1%) rated dietary advice provided by dietitians as vague. The most common complication experienced by the participants after bariatric surgery was nausea (61.4%). Furthermore, the majority of the participants (83.4%) found their daily and leisure activities to be more enjoyable after bariatric surgery. Ways of improving the quality of information delivery by dietitians should be explored to enhance patient comprehension and adherence to postbariatric surgery diet recommendations. Future research involving a larger and more representative sample to extend our findings are needed.

## 1. Introduction

According to the World Health Organization (WHO), at least 2.8 million people die every year because of being overweight or obese. Moreover, the worldwide prevalence of obesity has increased manifold between 1980 and 2014, with 13 percent of the world's adult population found to be obese during a survey conducted in 2014 [1].

Obesity has become a critical issue in the Arabian Gulf region [2]. According to the 2016 Global Health Observatory Report, the obesity prevalence among adults 18 years and above has tripled during 1975 to 2016 period in the Eastern Mediterranean Region [3]. In 2016, the prevalence of obesity in Kuwait was 37.9% and 31.7% in the United Arab Emirates (UAE) [3]. Obesity is a major public health concern in the United Arab Emirates (UAE). According to the Global Burden of Disease Study 2013 report, more than 66% of males, 60% of females, almost 40% of children aged 11 to 16 years, and 5% of children under the age of 11 in the UAE are obese [4]. A previous national study found 65% of adult women were either overweight or obese [5].

Obesity is clearly associated with increased morbidity and mortality. There is strong evidence that when obese individuals lose weight, the risks for type 2 diabetes and cardiovascular diseases (CVD) are reduced [6]. Weight loss strategies include dietary therapy, physical activity, pharmacotherapy, behavioral therapy, and bariatric surgery. Bariatric surgery is currently the most effective treatment modality for morbid obesity when compared with nonsurgical interventions and leads to improvement in medical comorbidities [7]. Candidates must have a body mass index (BMI) greater than 40 kg/m^2^ or a BMI greater than 35 kg/m^2^ with significant obesity-related disease in order to be eligible for bariatric surgery [6]. The total number of bariatric procedures performed worldwide in 2013 was 468,609, out of which 95.7% were carried out laparoscopically [8]. According to a global survey conducted by the International Federation for the Surgery of Obesity and Metabolic Disorders (IFSO) in 2013, Kuwait, one of the countries in the Arab Gulf region had the highest number of bariatric surgeries performed as a percentage of the national population, leading with 0.1642% [8].

Although bariatric surgery is an effective weight loss modality, patients with a history of bariatric surgery are at a greater risk of malnutrition and should be monitored for nutritional status [9, 10]. Types of malnutrition after surgery include protein-energy malnutrition and micronutrient deficiencies, such as iron, folate, vitamin A, and vitamin B_12_ [10]. Nutritional care is a vital component of the multidisciplinary healthcare before and after bariatric surgery to promote nutritional wellbeing and facilitate weight loss [11]. According to the guidelines published by the American Society for Metabolic & Bariatric Surgery, patients should undergo an appropriate nutritional evaluation, including micronutrient measurements, before any bariatric surgical procedure, as well as their ability in incorporating nutritional and behavioral changes before and after bariatric surgery [9]. Moreover, bariatric patients who do not adhere to recommended dietary guidelines postoperatively are at a greater risk in developing nutrition-related complications [12].

The countries in the Arabian Gulf region, such as Kuwait, Saudi Arabia, Qatar, and United Arab Emirates, have some of the highest rates of bariatric surgery, while having lower number of research and publications related to bariatric surgery outcomes compared to western countries with comparable bariatric surgery rates [13]. Moreover, there is limited research about the nutrition knowledge of bariatric postsurgery patients both globally and in the Arab Gulf region. In this pilot study, we explored nutrition knowledge related to postbariatric dietary recommendations among postbariatric surgery patients attending outpatient nutrition clinic of a major hospital in Dubai, United Arab Emirates. We also explored their satisfaction levels with dietitian counseling and decision to undergo bariatric surgery.

## 2. Materials and Methods

### 2.1. Research Design and Participants

In this cross-sectional pilot study, data were collected from postbariatric surgery patients attending an outpatient bariatric clinic of Rashid Hospital in Dubai, United Arab Emirates, during the period from July to September 2017. The sampling method used was a stratified random sample by gender whereby the sample sizes were proportional to the actual strata sizes in order to take into consideration any possible differences between both genders. The sample consisted of 70% females and 30% males in order to reflect the actual population gender distribution [14]. Based on hospital statistics of 336 female bariatric patients and 144 male bariatric patients per year, the required sample size to reflect the study population with an error of 10% was 58 females and 25 males (83 patients in total) which was calculated using Minitab. However, in our study, the actual collected sample of patients consisted of 29 males and 54 females. It is worthwhile mentioning that there have been no previous studies of similar nature to use as a reference in calculating the sample size. Eligible participants were UAE nationals aged 18–60 years who underwent bariatric surgery at Rashid Hospital, which is a governmental hospital that provides free healthcare services to UAE nationals including bariatric surgery.

The target age group was 18- to 60-year-olds since the majority of patients undergoing bariatric surgery are in this age range. Only patients who attended postsurgery follow-up visits with the dietitian and gave consent for their participation were recruited. In Rashid Hospital, as part of the routine care, all patients receive nutrition counseling after hospital admission before and after the surgery. Topics covered include general healthy eating guidelines, postsurgery diet progression, protein, and vitamin and mineral supplementation. In addition, management and prevention strategies of bariatric surgery related nutrition complications, such as dumping syndrome, nausea, vomiting, dehydration, and food intolerance, are discussed. Patients with BMI 50 kg/m^2^ or higher receive outpatient nutrition counseling and are advised to follow a calorie restricted diet to promote weight loss prior to the surgery. This information is based on international guidelines for the nutritional care of bariatric surgery patients [9, 15].

Potential participants were approached in the outpatient surgical and dietitian clinic, and the purpose of the study was explained to them. The research team approached 105 patients attending the outpatient and 83 of them agreed (response rate of 79%) to participate in the study. Both verbal and written informed consent was obtained from those who agreed to participate.

### 2.2. Data Collection

Data were collected through a questionnaire, which was developed by two dietitian members of the research team from the existing educational materials that dietitians provide to postbariatric patients, in the hospital where the study was conducted. The educational materials contain information about the dietary stages that postbariatric patients are expected to follow, the duration of each stage, and the type of food to include and avoid at each stage. The educational materials also include tips on how to avoid diet-related complications, such as vomiting, after bariatric surgery.

Since the questionnaire items were adopted from nutrition education materials in Arabic, and all patients were Arabic-speaking, the questionnaire was developed and administrated in Arabic. Three clinical dietitians from the hospital reviewed the final questionnaire for relevance before pretesting it with six postbariatric surgery patients. The questionnaire was modified based on the feedback from the participants by rewording some of the items for clarity and removing questions related to general nutrition knowledge. Although the questionnaire was self-administered, the principal investigator was available to address participants' questions and provide clarifications while completing the questionnaire.

The following topics were covered in the questionnaire: postbariatric dietary stage 1 (1 item); knowledge of diet recommendations after bariatric surgery (14 items); dietitian follow-up; clarity of information provided by the dietitian and adherence to the postbariatric diet recommendations (3 items); nutrition-related complications associated with bariatric surgery, including nausea, vomiting, and dumping syndrome (1 item); and participants' satisfaction with their decision to undergo bariatric surgery (2 items). Each correct answer to the questions related to knowledge of postbariatric surgery diet recommendations was assigned a score of 1 and incorrect answer a score of zero. Thus, the maximum possible knowledge score of bariatric dietary recommendations was 14. Participant's rating of the clarity of the information conveyed by the dietitian was evaluated on a scale of 1 to 10 (1 being very clear and 10 being vague). Participant's level of adherence to the dietitian recommendations was assessed using a scale of 1 to 10 (1 being very compliant and 10 being noncompliant).

### 2.3. Data Analysis

Data were analyzed using the Statistical Package for Social Science (IBM-SPSS, version 23.0). Descriptive statistics such as frequencies, proportions, means, and standard deviations were used. Correlations were established between certain sets of questions using either the chi-squared test of independence or Pearson's correlation, depending on whether it was a comparison between categorical or quantitative variables, respectively. The independent samples *t*-test and one-way ANOVA were used to compare the mean scores of different questions across two or more groups. The normality and the homogeneity of the variances of the response variables were tested using Shapiro–Wilk test and Levene's test, respectively. The mean knowledge score for each participant's response was calculated, and the total scores were divided into three categories: equal or less than 7, 8–11, and 12–14. We considered a score of 7 or less (50% or less) as low knowledge, 8–11 as a moderate knowledge, and 12 or more as sufficient knowledge about postbariatric surgery nutrition knowledge. For the analyses, a *p* value of <0.05 was considered statistically significant.

### 2.4. Ethical Consideration

Ethical approval for the study was obtained from Dubai Health Authority Research Committee (Protocol number: DSREC-SR-07/2017_01). All participants were informed that their participation in the project is voluntary and that all information collected will remain anonymous and confidential. Verbal and written consent was obtained from each participant prior to collecting any data.

## 3. Results

This study was conducted to explore nutrition knowledge of postbariatric surgery diet recommendations. Eighty-three postbariatric surgery patients aged 18 to 60 years (median age 40 years) attending the outpatient nutrition clinic of a major hospital in Dubai participated in the study. The majority of the participants (65.1%) were females (Table 1). In regard to the postbariatric dietary stage, 69.9% of the participants had resumed a regular diet, 13.3% were following a mashed diet, 10.8% were on a soft diet, and only 6% were on clear fluids as presented in Table 1. The majority of the participants (79.5%) reported adherence to the physician's instructions to take the daily vitamin/mineral supplements after surgery (Table 1).

Most of the participants, 78.3%, correctly identified cream soup as one of the foods, which is recommended to consume during the first stage of the postbariatric surgery diet (Table 2). The remaining 21.7% were not able to provide the correct response.

The results of the 14 questions used to assess postbariatric surgery diet knowledge of the participants are shown in Table 3. The participants' mean knowledge score (SD) about postbariatric diet was 9.7 (2.05). The minimum score was 4 and maximum was 14. More than 90% of the participants correctly stated the need to consume protein supplements prescribed by the dietitian in order to help in sparing muscle mass and assist the wound healing process. Furthermore, over 90% of the participants also correctly stated that, in the long-term, they would need to follow a diet low in fat, low to moderate in fiber, medium to high in protein, and low in simple sugars (Table 3). On the other hand, less than 50% were able to respond correctly to the questions related to drinking fluids from a straw to control the amount of fluids consumed, as well as choosing apple sauce instead of a whole apple during the third stage of the postbariatric diet.

Postbariatric surgery diet recommendations knowledge scores of the participants ranged from 4 to 14 with higher scores indicating greater knowledge of bariatric diet recommendations. The majority (66.4%) scored between 8 and 11. On the other hand, 14.4% of participants received a score of ≤7 and only 19.2% scored within the ≥12 range. The mean nutrition knowledge scores were compared in relation to the demographic data including gender and marital status. A significant difference was found between the total nutrition knowledge scores of male and female participants (*p* value = 0.02). The mean score for males was 9.0 ± 1.88 and for female participants 10.0 ± 2.05. However, there was no significant difference between the scores of married and single participants (*p* value = 0.313). Finally, there was no significant difference in the knowledge scores of subjects from the various dietary stages about postbariatric surgery dietary recommendations (*p* value > 0.1).

The majority of the participants (79.5%) reported that they regularly follow up with their dietitians, according to the hospital visit schedule for postbariatric surgery patients (Table 4). Regarding the clarity of the information provided by the dietitians to the patients, only 7.2% of them rated the clarity of the information conveyed by the dietitian, 1–3 (on a scale of 1 to 10, 1 being very clear and 10 being very vague). Moreover, when asked about their level of adherence to the dietitian instructions, only 10.8% of patients rated themselves in the 1–3 range (on a scale of 1–10, with 1 being very compliant and 10 being noncompliant) (Table 4). Moreover, there was a significant correlation (*p* < 0.001; *r* = 0.518) between the level of clarity of the dietitian's instructions and the patient's reported level of adherence to the postbariatric diet recommendations.

**Figure 1:**
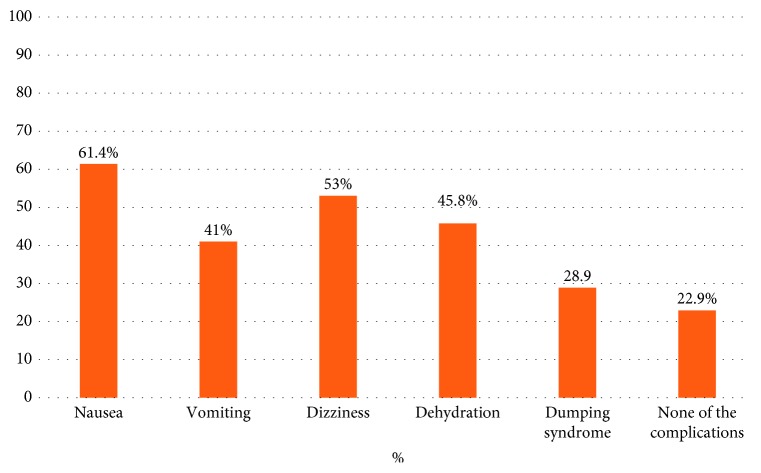
Nutrition-related complications reported by the participants.

The most common nutrition-related complications participants faced after bariatric surgery were also explored. The majority of the participants (61.4%) experienced nausea, followed by dizziness (53%), while 22.9% reported not experiencing any of the complications listed (Figure 1).

The final two questions of the questionnaire explored the participants' satisfaction level after undergoing bariatric surgery. Among the participants, 48.5% stated that they had not felt fatigued or regretted their decision to undergo bariatric surgery. However, 28.9% stated they had sometimes experienced fatigue or regretted their decision to undergo bariatric surgery. Only 6% of the participants stated that they always experience these negative emotions (Table 5). Regarding the respondents' satisfaction levels with their daily living and leisure activities, the majority of the participants (83.1%) found their activities to be more enjoyable than before surgery.

## 4. Discussion

The present study assessed the nutrition knowledge of bariatric patients attending an outpatient bariatric nutrition clinic in Dubai. Although the relationship between nutrition knowledge and dietary behaviors is conflicting [16–18], knowledge and health literacy are important determinants of dietary behaviors. The importance of nutrition education for bariatric surgery patients' postsurgical success has been documented in the literature [19–22].

In the present study, the majority of the participants correctly responded to the knowledge question related to the clear liquid diet stage. This information is usually given during the hospital stay and is most likely retained as a result of frequent daily reinforcement by the bariatric surgery management team. More than two-thirds of the participants reported moderate or low adherence to dietitian advice. This finding may be partly explained by social pressures, especially considering food-sharing practices during interactions with family and friends within the cultural context of the UAE. Although most participants in this study reported regular dietitian follow-ups, less than 10% of them rated the dietitian instructions as being of high clarity (1–3 rating). Not surprisingly, there was a strong positive correlation between the level of clarity of information conveyed and patients' compliance to prescribed diet. We recommend that dietitians include assessment of the patient's comprehension of the advice given as an integral part of the nutrition counseling. Moreover, dietitians need to develop better communication skills in order to improve clarity of the nutrition advice provided to postbariatric surgery patients. Although, the majority of the participants in the present study reported taking their daily vitamin-mineral supplements, additional efforts are needed to encourage all patients to take supplements daily, given their high risk for micronutrient deficiencies. On the other hand, it is encouraging to note that four out of every five of the participants reported regular follow-ups with their dietitian in the bariatric outpatient clinic. This is partly due to the scheduling of outpatient clinic appointments before patient discharge from the hospital, for better continuity of care.

Nausea and vomiting were the two most common complications, being experienced by over 50% of the patients. Both poor adherence to the postbariatric dietary recommendations and side effects of surgery can lead to nausea and vomiting [23].

The main limitation of this study was the cross-sectional design involving a relatively small number of patients, which limits the generalization of the results. With a larger sample size, participants could have been grouped according to various sociodemographic characteristics. This could provide better understanding about the association between postbariatric diet adherence and demographic factors. Female participants in the present study had significantly higher mean knowledge score compared to males. This finding is consistent with previous research indicating that women generally perform better than men do on tests related to nutritional knowledge and adoption of healthier dietary habits [24, 25]. Another limitation of the present study is the number of questions covered in each of the areas in the questionnaire. Future studies should examine these areas in detail by increasing the number of relevant questions. To the best of our knowledge, this is the first study that provides an important insight into the nutrition knowledge level of postbariatric surgery patients from one of the countries in the Arab Gulf States. The Arab Gulf region has some of the highest prevalence rates of obesity, as well as the number of bariatric surgeries performed as a percentage of their national populations [8]. Moreover, although few studies have evaluated educational interventions for bariatric surgery candidates [21, 22], ours is one of the first to evaluate bariatric surgery patient satisfaction with the quality of nutrition advice received from dietitians.

Currently, there are no validated questionnaires designed specifically for the assessment of nutrition knowledge after bariatric surgery in Arab Gulf region. Most of the existing instruments were developed to examine general nutrition knowledge [26–28], for a specific area within the field of nutrition (e.g., fat content and cholesterol content) [29] or were developed for non-Arabic-speaking populations [21, 22]. Therefore, there is a need to develop validated nutrition knowledge questionnaires for Arabic-speaking postbariatric populations.

## 5. Conclusions

The results of the present study have important implications in improving nutrition counseling provided to bariatric surgery as well as patient's adherence to the recommended dietary behaviors after the surgery. These implications include the need to develop and implement nutrition counseling strategies that improve clarity of the information delivered by dietitians to bariatric surgery patients as well identifying other ways to improve dietary adherence.

Further studies involving larger sample size and using validated questionnaires specific for assessing nutrition knowledge among bariatric surgery patients and their dietary behaviors for populations in the Arab Gulf region are warranted. Finally, future studies should explore in detail the nutrition knowledge of postbariatric patients and the reasons contributing to low adherence to dietitian nutrition advice as well as identify ways to improve dietary adherence.

## Figures and Tables

**Table 1:** Demographic characteristics of study participants.

	*n*	%
Gender		
Male	29	34.9
Female	54	65.1

Type of surgery		
Roux-en-Y gastric bypass	58	70
Sleeve gastrectomy	25	30

Dietary stage		
Clear fluids	5	6.0
Mashed diet	11	13.3
Soft diet	9	10.8
Regular diet	58	69.9

Marital status		
Married	51	61.4
Single	32	38.6

Are you compliant with taking your daily vitamin/mineral supplements?		
Yes	66	79.5
No	17	20.5

**Table 2:** Knowledge levels about the first stage postbariatric diet recommendation of the participants (*n* = 83).

	*n*	%
Which of these foods is not recommended to have during the first stage (clear fluids) after bariatric surgery?		
Cream soup	65	78.3
Clear soup	3	3.6
Chicken stock	11	13.3
Vegetable stock	4	4.8

**Table 3:** Knowledge of postbariatric surgery diet recommendations.

	Items	Correct response	
		*n*	%
1.	It is recommended to resume drinking coffee after the surgery as soon as you are allowed to eat and drink (false)	49	59.0
2.	In stage two (full fluids), you can have a fruit juice with pulp (false)	41	49.4
3.	In stage two (full fluids), you can have a full fat pudding or custard (false)	54	65.1
4.	You should drink your fluids using a straw to control amount consumed (false)	35	42.2
5.	You should drink a minimum of 1 litre of water in the first two weeks after the surgery (true)	69	83.1
6.	In stage 3 (mashed diet), you can eat scrambled eggs but not boiled eggs (true)	58	69.9
7.	You should limit your portion size by having your own set of small plates and bowls (true)	74	89.2
8.	You can drink fluids directly before eating food (false)	63	75.9
9.	Main meals should not exceed 3/4 of a cup and snacks should be within 1/4 to 1/2 of a cup (true)	65	78.3
10.	It is recommended to have an apple sauce instead a whole apple during stage 3 of the postbariatric diet (true)	34	41.0
11.	Which of the four cheeses, cheddar, akkawi, halloumi, and cottage cheese, is considered to be the lowest in fat and is suitable for postbariatric patients? (cottage cheese)	50	60.2
12.	It is advisable to have low-fiber diet after the surgery in order to prevent blockage (true)	57	68.7
13.	It is advisable to have a high-protein diet by consuming the protein supplements prescribed by the dietitian in order to help in sparing your muscle mass and the wound healing process (true)	77	92.8
14.	The diet you should follow bariatric surgery on the long-term basis should be low in fat, low to moderate in fiber, medium to high in protein, and low in simple sugars (true)	76	91.6

**Table 4:** Perceived importance of dietitian follow-up, clarity of dietitian's instructions, and level of compliance (*n* = 83).

	*n*	%
Do you follow up with your dietitian on regular basis?		
Yes	66	79.5
No	17	20.5

Rate your compliance with the postbariatric surgery diet recommendation (1 being very compliant, 10 being noncompliant).		
1–3	9	10.8
4–7	35	42.2
8–10	39	47

Rate the clarity of information conveyed by the dietitian (1 being very clear, 10 being vague)		
1–3	6	7.2
4–7	18	21.7
8–10	59	71.1

Compliance: 1–3 (high); 4–7 (moderate); 8–10 (low). Clarity: 1–3 (high); 4–7 (moderate); 8–10 (low).

**Table 5:** Satisfaction levels of postbariatric surgery patients (*n* = 83).

	*n* (%)
How often do you feel fatigued or regret your decision to have bariatric surgery?	
Never	38 (45.8)
Sometimes	24 (28.9)
Rarely	16 (19.3)
Always	5 (6.0)

How do you now find your daily and leisure activities?	
More enjoyable	69 (83.1)
Less enjoyable	3 (3.6)
I did not notice any difference	11 (13.3)

## Data Availability

The data used to support the findings of this study are available from the corresponding author upon request.
